# Two Cases of the Successful Termination of Wounds That Have Been Hitherto Deemed Mortal; with Observations

**Published:** 1800

**Authors:** W. Simmons

**Affiliations:** Member of the Corporation of Surgeons of London; and Senior Surgeon to the Manchester Infirmary.


III. *Two Caſes of the ſucceſsful termination of Wounds that have been hitherto deemed mortal; with Obſervations. By the Same.*

### CASE I.

*Diviſion of the internal jugular vein.*

ELIZABETH CLOUGH, aged 28 years, was admitted, under my care, into the Mancheſter Infirmary, in the year 1791, for a large tumour on the left ſide of the neck, extending from the outer edge of the ſterno-cleido-maſtoideus muſcle nearly to the articulation of the ſhoulder. From the appearance reſembling cancer, and it being occaſionally painful but moveable, it was determined, in confultation, to attempt the immediate extirpation.

I began the operation by a circular incifion through the common integument, and then grafping the tumour firmly in my left hand I diffected it out from the fubjacent parts. The divided arteries bled freely, but the blood rufhed in a torrent from the internal jugular vein, which had been divided in the operation. My affiftants compreffing the divided veffels they were alternately fecured, and laftly, the jugular; to fafely accomplifh which three ligatures were neceffary. The wound was now dreffed in the cuftomary way; every, even the flighteft, exertion was forbidden; and opiates were adminiftered, from time to time, fo as to procure eafe, and, if poffible, prevent any irritation from affecting the trachea or noftrils. The ligatures came away in due time, without any difpofition to hemorrhage manifefting itfelf, and the parts healed in about two months.

Chirurgical writers have claffed a wound of the internal jugular vein in the lift of mortal wounds; and, I believe no inftance is recorded of it being thus fecured and the patient furviving. Simple wounds of the neck are attended tended with much hazard, and, what is yet problematical, the danger ſhews itſelf, when, from the proſperous courſe of the diſeaſe, there is leaſt reaſon for apprehenſion.

The ſituation of this tumour preſented many difficulties. I felt confident of not injuring the carotid artery, or the par vagum and intercoſtal nerves, as they paſs along the neck; but was not entirely at eaſe reſpecting the right ſubclavian artery, the tumour dipping conſiderably under the upper edge of the clavicle. The coraco-hyoideus muſcle was expoſed through two thirds of its length. It may be proper to obſerve that I was compelled to remove the whole of the integument covering the tumour, it having become diſeaſed and adhering cloſely. The torrent of dark blood pouring out from ſo large a cavity, and the noiſe occaſioned by the ruſhing in of air, added to the other appearances, formed a picture more frightful than any I ever beheld. Such caſes, fortunately, do not often occur; but, in this melancholy and almoſt hopeleſs condition, ſome conſolation may ariſe to the patient, and an acceſſion of fortitude may be derived to the ſurgeon, from the recital of this caſe. In tumours thus ſituated the danger muſt be imminent to juſtify an operation; ſhould the patient, however, ſolicit it, after a candid explanation of the doubtful event the ſurgeon will feel it his duty to perform it. While, on the one hand, timidity conſigns the patient to the hopeleſs and cruel ravages of an incurable diſeaſe; on the other, circumſpection and a nice diſcrimination of the attendant circumſtances will be neceſſary to enable the ſurgeon to proſecute, with ſucceſs, the only expedient for the preſervation of life.

She experienced no morbid affection of the head from the obliteration of this veſſel, and the parts would have healed in a much ſhorter time, but for the cancerous diſpoſition ſhewing itſelf, when they were nearly cicatrized; which yielded in this form to the external application of arſenic. The cancer had ſo connected itſelf with the jugular as to render it impracticable to remove the whole without dividing the vein.

### CASE II.

### *Wound of the Uterus.*

Ann Calvert, a married woman, 42 years of age, had laboured under an aſcites for eight years.

years. She had been under the care of doctor Ferriar for some time, without benefit, and was transferred to me to be tapped. On the 11th of April, 1799, I drew off about fourteen quarts of water, by introducing the trocar midway between the navel and pubes; * the urgent symptoms were relieved, but on a second collection taking place I was desired to tap her again. The second operation was performed on the 1st of July following, when instead of water I was alarmed with a discharge of blood; which, after being permitted to flow to the quantity of about six ounces, ceased on withdrawing the canula. As suspicions had arisen, previous to this tapping, that she was with child, I directed an opiate, and enjoining rest, predicted an unfavourable termination; her breasts were enlarged; she had seen nothing of the catamenia for several months; and the fluctuation was so obscure, it was only on her positive assurance to the contrary, and from having drawn off so much water before, that I could be prevailed on to tap her, though she came into the Infirmary

* See Doctor Ferriar's Med. Hist. and Reflect. vol. I. page 43.

voluntarily voluntarily for that purpofe. The event fhewed that fhe was miftaken, and fhe was delivered of a fine healthy girl on the 26th of October, having fuffered no inconvenience from the laft operation. The accumulation again recurring I tapped her for the third time on the 29th of Auguft, 1798, and drew off ten quarts and half a pint of ftraw-coloured fluid, coagulable by heat. Particular care was taken to make this perforation clofe to the former, yet there was no difcharge of blood, and I obferved with fatisfaction, that the enlarged vifcera were decreafed in fize. She was relieved by this operation, and no unpleafant confequence enfued.

A wound of the uterus has been ranked likewife in the lift of mortal wounds, and it would appear juftly, from the termination of all the cafes of the Cæfarean operation that have been performed in this country. In other countries it has been more fuccefsful. Sonnius, a phyfician at Bruges, we are told, performed it feven times on his own wife, with fuccefs both to the mother and child. However improbable this ftory may appear, it is admitted, on undoubted teftimony, to have been performed with fuccefs on the continent, under circumftances circumſtances not likely to promote it, either from the ſkill or dexterity of the operator. To what this difference of reſult is owing, whether to a difference of climate or to other cauſes, does not now appear to be of much moment, ſince, from the able manner in which the ſubject has been treated by Doctor Oſborn, it would ſeem to be unjuſtifiable during the life of the mother.

Relative to this woman it may be obſerved, that ſhe was five months gone with child at the ſecond, and between two and three months at the time of the firſt operation; clearly evincing that the operation of the paracentiſis may be performed, in the early months of pregnancy, to relieve urgent ſymptoms, with perfect ſafety. A fact of great practical import, conſidered in relation either to the mother or child; for if, in addition to the diſtention of the abdomen by water, the womb ſhould go on increaſing to the full period of geſtation, a rupture might be apprehended; and from the continual preſſure on the womb, it is not unlikely that abortion or premature labour would be produced. An additional hazard will be incurred alſo by the mother, from the diſtention preventing the diaphragm and abdominal mufcles affording any help in time of labour, fo that the expulfion of the child muft reft folely on the action of the uterus There was a confiderable collection of water at the time of the fecond operation; but, from the fize of the abdomen after delivery, and the length of time that elapfed before the third became neceffary, it would feem that the depofition had been fufpended in the latter months, and for fome time after delivery. It is indifputable that pregnancy checks the progrefs of phthifis pulmonalis; hence it may be warrantable to conclude, in the prefent cafe, that the uterine irritation, operating in a fimilar way, arrefted the progrefs of the difeafe; and after delivery, that the diverfion to the breafts would ftill counteract the morbid tendency. She had a copious fecretion of milk, and fuckled her child for feveral months. Should this be the ufual courfe, the danger from the caufes above recited will be much leffened; and I fhall be glad to find it confirmed by future experience.

In tapping I have often feen the trocar driven up to the reft of the canula, with a dexterous violence that has ftruck me with dread, I have therefore made it a rule not to puſh it farther than juſt through the parietes, which is ſufficient for every purpoſe of drawing off the water; and this caſe will ſerve to ſhow what would have been the probable conſequence of a contrary practice. The laſt operation proves deciſively that the blood came from the uterus; it flowed out in a conſiderable ſtream, but ceaſed, as before-mentioned, on withdrawing the canula, the lips of the wound no doubt immediately cloſing. The pain ſhe ſuffered at the time was more than usual, yet not ſo great as to excite any alarm, and the ſoreneſs after was little more than what ſhe had formerly experienced. It had the appearance of venous blood, without any admixture of water; had the inſtrument penetrated ſo deep as to have diſcharged the waters, it may be reaſonably inferred that ſhe would have miſcarried. My preference of this mode of operating will be admitted from what I have formerly offered on the ſubject; in the preſent caſe there was no alternative, from the enlarged ſtate of the viſcera on each ſide.

